# Retraction: Genetic Variants in DNA Double-Strand Break Repair Genes and Risk of Salivary Gland Carcinoma: A Case-Control Study

**DOI:** 10.1371/journal.pone.0199690

**Published:** 2018-06-21

**Authors:** The PLOS ONE Editors

Following publication of this article [1], the authors discovered that many subjects in the study had identical genotypes, implicating that the sampling had been compromised with multiple duplicate samples; this affected 117 controls and 4 cases. This concern was subsequently investigated by the U.S. Department of Health and Human Services' Office of Research Integrity (ORI). The ORI found evidence that a contributor to this study who was involved in participant recruitment and sample collection had falsified data and samples, "by recording dates and providing her own blood samples to cause these samples to be falsely labeled" [2]. This contributor is not a listed author on the article.

The falsified data and samples significantly impact the validity of the data analyses and results reported in the article. In light of these concerns, in line with the ORI's recommendation, and by agreement of the authors, the *PLOS ONE* Editors retract this article.

LX, HT, AKE, PW, and EMS agree with the retraction.
